# The night shift brain: functional network reorganization in sleep-deprived medical staff

**DOI:** 10.3389/fnhum.2026.1757604

**Published:** 2026-03-11

**Authors:** Zhen Zeng, Dingbo Guo, Liuheng Liu, Fangyuan Ou, Tingting Du, Lisha Nie, Hua Yang, Cong Peng

**Affiliations:** 1 The Department of Radiology, Chongqing Hospital of Traditional Chinese Medicine, Chongqing, China; 2 GE Healthcare, MR Research, Beijing, China

**Keywords:** total sleep deprivation, resting-state functional MRI, graph theory, medical staff, cognitive function

## Abstract

**Background:**

Medical staff frequently experience sleep deprivation, impacting both their health and patient care quality. Understanding brain network changes under sleep deprivation can guide preventive strategies. This study aims to determine how total sleep deprivation (TSD) alters brain network topology in medical professionals.

**Methods:**

Using graph-theory analysis of resting-state fMRI data from 36 medical staff, we assessed global and local brain network properties following TSD and normal sleep (rested wakefulness, RW), examining topological changes and their correlation with cognitive performance.

**Results:**

Small-world properties were present in both conditions, but the TSD condition showed higher clustering coefficients (*p* = 0.044). Key nodal changes included increased degree centrality in the right superior medial frontal gyrus (*p* = 0.0006) and decreased nodal efficiency in the left fusiform gyrus (*p* = 0.0004). Using the right superior medial frontal gyrus as ROI, enhanced functional connectivity (zFC) was observed in multiple bilateral frontal/temporal regions (peak *t* > 4.5). These topological changes correlated with cognitive deficits: reduced Digit Symbol Test (DST) scores (*p* < 0.001), prolonged Number Connection Test-A (NCT-A) and Line Tracing Test (LTT) completion times (*p* < 0.05), while increased clustering coefficients (Cp) positively correlated with NCT-A/SDT performance changes (*r* = 0.341–0.411, *p* < 0.05). And older staff exhibited greater vulnerability in global network efficiency and path length (*r* = −0.352, *r* = 0.390, *p* < 0.05).

**Conclusion:**

By identifying key brain network nodes affected by TSD, this study provides insights into neural adaptations under TSD, offering an evidence-based framework for developing both therapeutic interventions and preventive strategies to mitigate cognitive and health impacts in high-risk populations.

## 1 Introduction

Sleep serves as a fundamental pillar for maintaining physical and psychological well-being. Both sufficient duration and restorative quality of sleep are essential for the proper functioning of virtually all physiological systems. Inadequate or disrupted sleep can adversely affect a wide range of health domains, including cardiovascular function, emotional regulation, cognitive performance, and memory formation (Baranwal et al., 2023). Sleep deprivation (SD), a state of sleep loss, is classified into two distinct types - total sleep deprivation (TSD) and partial sleep deprivation (PSD), depending on the length of SD time. TSD is defined as staying awake for at least 24 h continuously, and PSD is defined as continuous or intermittent sleep that is less than 50% of normal sleep (Reynolds and Banks, 2010). SD has been shown to have negative effects on memory, cognition and metabolism (Killgore, 2010; Davies et al., 2014). It is worth noting that, due to demanding work schedules, medical staff often suffer from SD, which may lead to both physiological and cognitive deficits and further impacting their overall health, resulting in a decrease in the quality of medical care they provided (Neville et al., 2017; Johnson et al., 2014; Yan et al., 2023). Therefore, the potential harm of SD to medical staff is well worth studying. Investigating such state-dependent brain-behavior relationships aligns with contemporary research paradigms that employ large-scale, ecologically valid designs to uncover neural pathways linking environmental factors with mental health (Yu et al., 2025).

Although many studies have investigated the changes in brain function after SD, the precise mechanism requires further investigation. Resting-state functional magnetic resonance imaging (rs-fMRI) provides a non-invasive tool to probe this mechanism. Applying graph theory—a discipline for studying network topology—to rs-fMRI data allows the brain to be conceptualized as a complex network. This offers a mathematical foundation for quantifying its connectome’s topology, thereby illuminating the structural basis of functional impairments and providing diagnostic and therapeutic insights (Miraglia et al., 2016). This method is now widely used in studying brain disorders like Alzheimer’s disease (Frantzidis et al., 2014) and schizophrenia (Anderson and Cohen, 2013). Large-scale, reproducible neuroimaging studies have further established reliable brain-behavior associations, demonstrating for instance that cortical surface area reductions are linked to the general psychopathology factor in adolescents, highlighting the utility of structural metrics as transdiagnostic biomarkers (Kuang et al., 2023).

Luo et al. (2024) observed a notable decrease in modularity alongside an increase in global efficiency within brain networks following SD. This pattern contrasts with findings from some studies, which reported reductions in both parameters (Yeo et al., 2015), but aligns with other research that also documented elevated global efficiency post-SD (Liu et al., 2014). The above researches had indicated that changes of spontaneous brain activity manifested after TSD, while most of them were conducted under controlled laboratory conditions. Departing from the norms of daily practice, the night shift requires medical staff to operate in a frenetic and high-pressure environment, managing a distinct set of challenges that markedly diverge from the controlled conditions of experimental TSD studies. Consequently, the specific effect of TSD on the brain functional network topology of medical staff merits dedicated investigation. Previous findings from our team demonstrated that TSD in medical staff led to widespread changes in several metrics of regional brain activity and connectivity: the amplitude of low-frequency fluctuations (mfALFF), regional homogeneity (zReHo), and functional connectivity (zFC) (Peng et al., 2024).

Based on these localized alterations, we hypothesized that TSD would also induce reorganization at the whole-brain network level. Specifically, we aimed to test whether TSD alters global topological properties (e.g., small-worldness, efficiency) and redistributes centrality among key nodes. To test these hypotheses and further elucidate the network-level organization underlying these regional changes, this study leverages graph-theory analysis alongside FC methods to investigate the reorganization in global and local attributes of the brain functional network in medical staff post-TSD, providing insight into the deeper understanding of compensatory mechanisms and potential targets for intervention.

## 2 Methods

### 2.1 Participants and assessment

This study included 39 medical professionals (17 female; 22 male), comprising both doctors, medical technicians and nurses (15/14/7), with a mean age of 31.85 ± 4.36 years (range: 26–42). All participants worked a rotating schedule that regularly included night shifts, typically ranging from 2 to 4 per month, with the remaining days consisting of normal sleep periods. Participants were included according to the following criteria: (1) no prior diagnosis of neurological or psychiatric disease; (2) absence of clinically significant sleep disorders; (3) free from histories of tobacco smoking, alcoholism, or substance dependence; (4) no contraindications for MRI scanning, such as claustrophobia; (5) adherence to a normative sleep–wake cycle, characterized by a minimum of 6.5 h of nocturnal sleep, with bedtimes before 1:00 a.m. and wake times before 9:00 a.m. when not working night shifts (Chee et al., 2011). Ethical approval for this study involving human participants was granted by the Ethics Committee of Chongqing Hospital of Traditional Chinese Medicine (IRB approval no.: 2023-KY-KS-PC). Informed consent was obtained in writing from all individuals before their participation. For the final dataset, three participants were excluded due to either excessive head motion (one subject) or failure to complete the follow-up assessment (two subjects).

The impact of TSD on cognitive functions was evaluated using the following neuropsychological tests: the Number Connection Test-A (NCT-A), Digit Symbol Test (DST), Line Tracing Test (LTT), and Serial Dotting Test (SDT) (Li, 2013). Each neuropsychological test targeted specific cognitive domains. Participants’ attention, mental flexibility, and psychomotor speed were evaluated using the NCT-A, which requires the rapid sequential connection of numbers. The DST served as an indicator of cognitive processing speed, attention, and working memory, with scores reflecting the number of symbols correctly matched to numbers under time pressure. Performance on the LTT, which involves accurately tracing lines, reflected individuals’ visuospatial ability and motor coordination. The SDT, requiring the connection of dots in a specific order, was employed to assess fine motor skills and hand-eye coordination (Peng et al., 2024). For all tests, a lower score was associated with improved performance, with the exception of the DST, for which a higher score was favorable.

All subjects received two MRI scans alongside neuropsychological testing, with the two sessions spaced at least 14 days apart. During the RW session, participants were tested in the morning after a night of normal sleep at home, immediately before starting a scheduled night shift. During the TSD session, participants were tested in the morning after having remained awake for approximately 24 continuous hours, which included working the preceding full night shift. Participants were randomly assigned to one of two conditions. All underwent a training session prior to scanning to minimize practice effects and acclimatize them to the MRI environment. Half of the participants began with the rested wakefulness (RW) scan, while the other half started with the total sleep deprivation (TSD) scan. The neuropsychological test battery was administered immediately prior to each MRI scan by a trained research assistant in a quiet room adjacent to the scanner suite.

### 2.2 MRI data acquisition

All MRI data were obtained on a 3.0 T GE SIGNATM Architect scanner (GE Healthcare, Milwaukee, WI, USA) equipped with a 48-channel head coil. For the BOLD-fMRI acquisition, a gradient-echo echo-planar imaging (EPI) sequence was employed with the following parameters: repetition time (TR)/echo time (TE) = 2000/30 ms, flip angle (FA) = 90°, 34 contiguous slices (interleaved order, 5 mm thickness), field of view (FOV) = 220 × 220 mm^2^, matrix size = 64 × 64, and 240 total volumes.

### 2.3 Data preprocessing

The preprocessing pipeline for the fMRI data were performed using the Gretna software (Wang et al., 2015).^1^ The pipeline, included the following key steps. First, the initial 10 volumes of each scan were discarded to allow for magnetization equilibrium. Subsequent steps included slice timing correction (using the middle slice as reference), head motion realignment (participants with motion exceeding 3.0 mm translation or 3.0° rotation in any direction were excluded), and spatial normalization to the standard Montreal Neurological Institute (MNI) space using the EPI template. Normalized images were resampled to 3 × 3 × 3 mm^3^ voxels and smoothed with a 6-mm full-width at half-maximum Gaussian kernel. Nuisance covariates, including the Friston 24-parameter model of head motion and signals from white matter and cerebrospinal fluid, were regressed out. Finally, a temporal band-pass filter (0.01–0.08 Hz) was applied to the time series to reduce low-frequency drift and high-frequency noise.

### 2.4 Graph theory network analysis

FC matrices were constructed based on the AAL atlas (Landeau et al., 2002) comprising 90 cortical and subcortical regions. For each participant, a 90 × 90 symmetric matrix was generated by computing Pearson correlation coefficients between the mean time series of every pair of regions. These correlation matrices were subsequently Fisher-z transformed to improve normality and stabilize variance for subsequent analyses.

To perform graph-theoretical analysis, binary undirected networks were derived by applying a proportional thresholding approach. This was done across a continuous sparsity (or cost) range from 0.10 to 0.22, with an incremental step of 0.01. At each sparsity level, the top corresponding percentage of connections (edges) with the highest absolute correlation values was retained, setting all other potential connections to zero. This process generated a series of binarized networks across the specified cost range.

Global network metrics (including the clustering coefficient, characteristic path length, normalized clustering coefficient, normalized path length, small-world index, global efficiency, and local efficiency) and nodal metrics (degree centrality and nodal efficiency) were calculated for each binary network at every threshold. To obtain a summary measure that is robust to the choice of a single arbitrary threshold and to integrate information across the entire cost spectrum, the area under the curve (AUC) for each metric was computed over the sparsity range of 0.10–0.22. These AUC values were then used as the primary metrics for all between-condition (RW vs. TSD) statistical comparisons.

Additionally, to examine the robustness of network construction, we repeated the binary network analysis using an extended sparsity range of 0.05–0.25 (step = 0.01) and weighted networks.

### 2.5 Statistical analysis

Clinical and demographic data were analyzed using SPSS 22.0 (IBM Corp., Armonk, NY, USA). The normality of distribution for all primary outcome variables was assessed using the Shapiro–Wilk test. To assess the effects of total sleep deprivation, paired-sample *t*-tests were employed for comparisons of global network metrics between the RW and TSD conditions. For analyses involving multiple parallel comparisons, false discovery rate (FDR) correction was applied to control for Type I error, a standard practice in large-scale neuroimaging studies to balance sensitivity and specificity (Yu et al., 2023). Specifically: For comparisons of nodal metrics (90 nodes), FDR correction was applied across all nodes for each metric separately. For seed-based whole-brain FC analyses, FDR correction was applied across all voxels within the whole-brain mask. A significance threshold of *p* < 0.05 (two-tailed) was adopted for all tests, with FDR-corrected *p*-values denoted as pFDR < 0.05. Spearman’s correlation was utilized to examine the relationships between alterations in neuropsychological test scores and changes in global network metrics.

In line with contemporary methodological standards for enhancing reproducibility (Fan et al., 2023; Yuchao Jiang et al., 2023), effect sizes for significant comparisons are reported alongside *p*-values. For parametric tests (paired *t*-ests), Cohen’s d with 95% confidence intervals (CI) is provided. For non-parametric tests (Wilcoxon signed-rank tests), the effect size *r* with 95% CI is reported. All confidence intervals were computed using standard methods for paired designs. Our reporting of effect sizes adheres to contemporary standards for transparency in neuroimaging research, aligning with methodological practices in large-scale, reproducible studies where careful consideration is given to effect size estimation within advanced statistical frameworks (Wu et al., 2025). Given the exploratory nature of these correlation analyses, we report effect sizes (*r*) with 95% confidence intervals to emphasize the estimation of association strength and precision. To validate the key brain-behavior associations identified in the exploratory correlation analyses, we performed linear regression analyses with the change scores of neuropsychological tests (ΔDST, ΔNCT-A, ΔLTT, ΔSDT) as dependent variables, the relevant network metric as the core predictor, and age and sex as covariates. Unstandardized coefficients (B) with 95% confidence intervals, standardized coefficients (*β*), *t*-values, and *p*-values were reported. Multicollinearity was assessed using variance inflation factor (VIF), with VIF < 5 considered acceptable.

## 3 Results

### 3.1 Demographic characteristics and cognitive performance

This study ultimately included a cohort of 36 participants (15 female; mean age 31.97 ± 4.29 years). Demographic characteristics and neuropsychological test results are summarized in Table 1. Cognitive performance was significantly impaired following TSD. Specifically, subjects exhibited a markedly lower score on the DST in the TSD state compared to the RW state (*t* = 4.182, *p* < 0.001, Cohen’s d = 0.70, 95% CI [0.34, 1.05]). Conversely, performance on the NCT-A (z = −2.35, *p* = 0.019, *r* = 0.39, 95% CI [0.07, 0.71]) and the LTT (*z* = −2.29, *p* = 0.02, *r* = 0.38, 95% CI [0.06, 0.70]) was significantly worse, as indicated by higher scores after TSD. Additionally, the SDT score also increased following sleep deprivation, although this difference did not reach statistical significance.

**Table 1:** Demographic and clinical data.

Parameters	TSD (*n* = 36)	RW (*n* = 36)	*t/z* value	*p* value	Effect size (d [95% CI])
Sex (M/F)	21/15	21/15	-	-	
Age (years)	31.97 ± 4.29	31.97 ± 4.29	-	-	
DST	64.81 ± 8.93	69.58 ± 9.43	4.18	<0.001^a^	0.70 [0.34, 1.05]
NCT-A (sec)	31.86 ± 8.70	29.28 ± 9.56	−2.35	0.019^b^	0.39 [0.07, 0.71]
LTT (sec)	54.69 ± 23.76	49.06 ± 17.32	−2.29	0.02^b^	0.38 [0.06, 0.70]
SDT (sec)	42.44 ± 8.54	41.33 ± 9.89	−1.71	0.087^b^	

TSD, total sleep deprivation for 24 h; RW, rested wakefulness after normal sleep. Paired samples *t*-test for continuous variables with a normal distribution, Wilcoxon Signed Rank test for continuous variables with a non-normal distribution. DST, Digit Symbol Test; NCT-A, Number Connection Test-A; LTT, Line Tracing Test; SDT, Serial Dotting Test.

^a^Paired samples *t*-test. ^b^Wilcoxon Signed Rank test.

### 3.2 Changes in rs-fMRI features between RW and TSD condition

#### 3.2.1 Between-condition comparison in global network metrics

Across a sparsity threshold ranging from 0.1 to 0.22 (Zhang et al., 2011), both the TSD and RW conditions displayed small-world network characteristics. The clustering coefficient was higher in the TSD condition than in the RW condition (*p* = 0.044, Cohen’s d = 0.35, 95% CI [0.01, 0.69], Table 2).

**Table 2:** Between-condition comparison in global network metrics.

Metric	TSD	RW	*t* value	*p* value	Effect size (d [95% CI])
Eglob	0.056 (0.003)	0.056 (0.002)	−0.551	0.58	
Eloc	0.870 (0.003)	0.861 (0.004)	1.267	0.213	
Cp	0.685 (0.003)	0.671 (0.003)	2.089	0.044	0.35 [0.01, 0.69]
Lp	0.263 (0.158)	0.262(0.014)	0.477	0.636	
γ	0.265 (0.462)	0.256(0.049)	0.895	0.376	
λ	0.140 (0.006)	0.140 (0.005)	0.248	0.806	
σ	0.227 (0.45)	0.219 (0.047)	0.785	0.438	

All data are represented by mean (standard deviation). Eglob, global efficiency; Eloc, local efficiency; Cp, clustering coefficient; Lp, characteristic path length; γ, normalized clustering coefficient; λ, normalized characteristic path length; σ, small - world index.

#### 3.2.2 Regions showing changed local network metrics between TSD and RW

**Figure 1:**
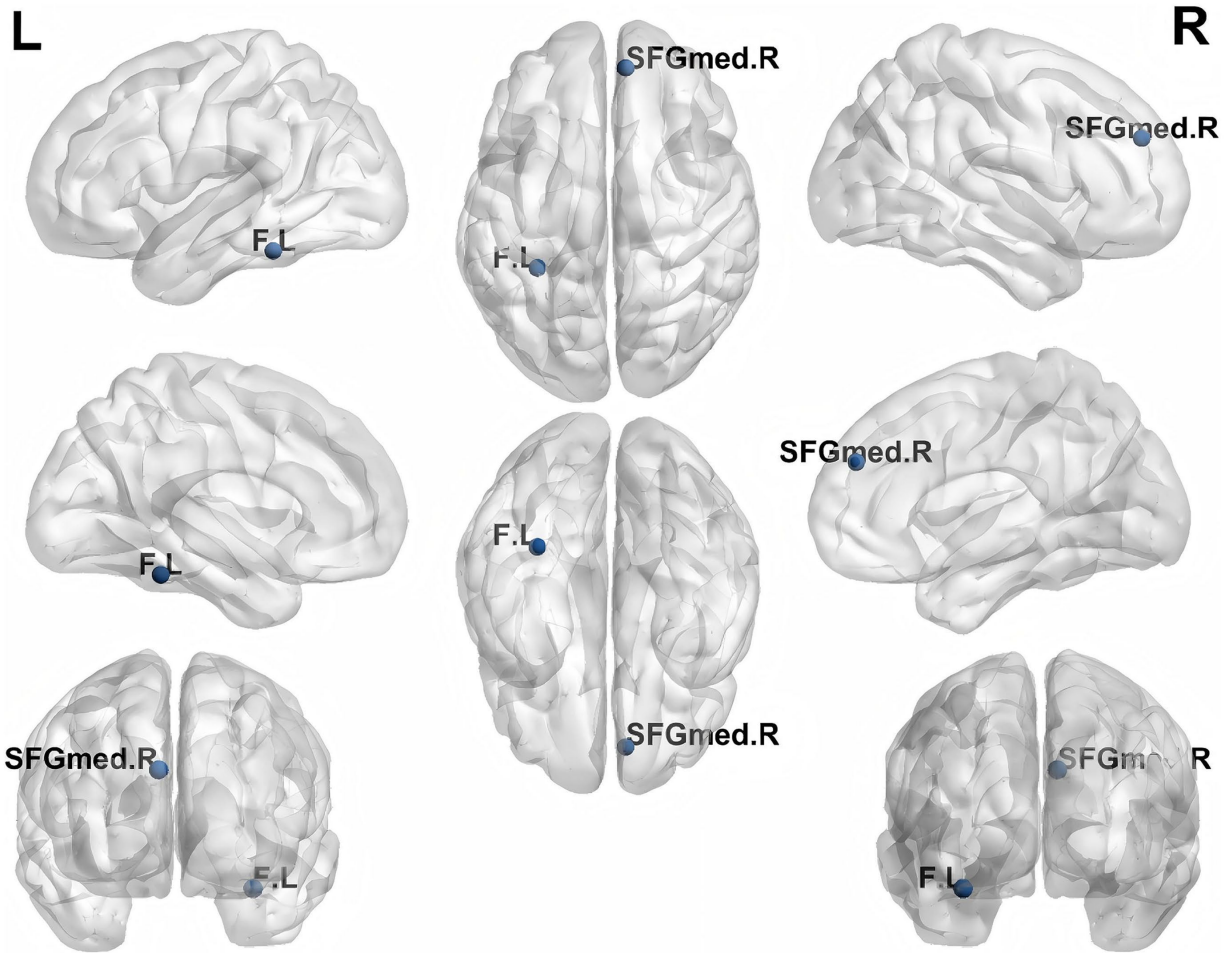
Brain regions with significant differences in node attribute changes in TSD conditions. Black balls represent the areas of changed local network metrics. SFGmed. R, right superior medial frontal gyrus F. L, left fusiform.

Degree centrality in the right superior medial frontal gyrus increased (*p* = 0.0006, Cohen’s d = 0.62, 95% CI [0.27, 0.98]), while nodal efficiency in the left fusiform gyrus decreased (*p* = 0.0004, Cohen’s d = −0.64, 95% CI [−0.99, −0.30]) in the TSD condition (Figure 1; Table 3).

**Table 3:** Regions showing changed local network metrics between TSD and RW.

Region	Hemisphere	AAL area	*p*-value	*t*-value	Effect size (d [95% CI])
Degree centrality					
superior medial frontal gyrus	R	24	0.0006	3.7454	0.62 [0.27, 0.98]
Nodal efficiency					
fusiform	L	55	0.0004	−3.8652	−0.64 [−0.99, −0.30]

Paired samples *t*-test was used to compare the differences between TSD and RW; AAL, Automated Anatomical Labeling atlas’s area.

#### 3.2.3 zFC changes between RW and TSD states

**Figure 2:**
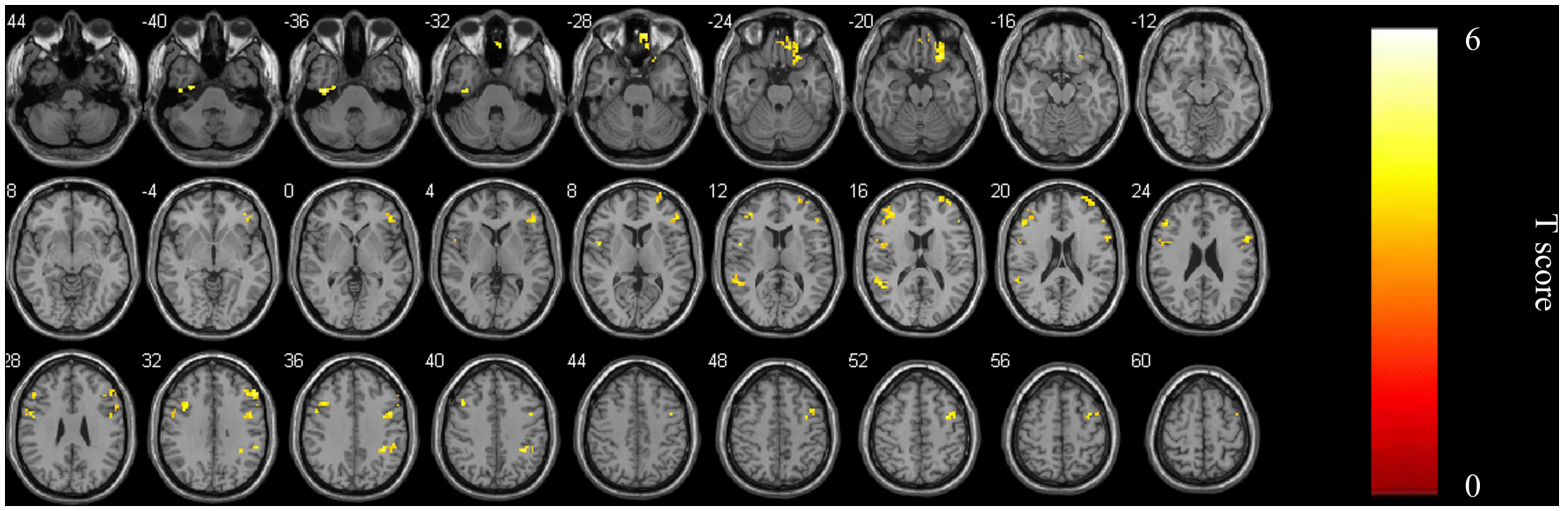
zFC of multiple brain regions of bilateral frontal and temporal gyrus enhanced after TSD using right superior medial frontal gyrus as ROI.

zFC was elevated in multiple bilateral frontal and temporal regions using the right superior medial frontal gyrus as the region of interest (ROI) (Figure 2; Table 4).

**Table 4:** Regions showing zFC differences between TSD and RW.

ROI	Brain regions	Side	AAL	Voxels with the maximum effect				
				MNI (x, y, z)			voxels	*t*-value
ROI	Inferior temporal gyrus	L	89	−39	−18	−36	26	6.155
	Rectal gyrus	R	27	9	54	−27	39	4.9342
	Inferior orbital frontal gyrus	R	16	24	27	−21	59	5.2631
	Inferior triangular frontal gyrus	R	13	48	36	6	49	4.915
	Inferior opercular frontal gyrus	L	11	−51	6	6	57	5.0865
	Superior frontal gyrus	R	4	36	57	18	43	4.6021
	Superior temporal gyrus	L	81	−54	−45	15	44	4.9921
	Middle frontal gyrus	L	7	−39	45	18	66	4.5203
	Inferior opercular frontal gyrus	R	12	54	12	27	23	4.5361
	Middle frontal gyrus	R	8	35	0	51	78	4.7918
	Middle frontal gyrus	R	8	51	27	33	37	5.0784
	Supramarginal gyrus	R	64	36	−42	39	28	4.7652
	Middle frontal gyrus	L	7	−36	18	33	29	4.5387

Paired samples *t*-test was used to compare the differences between TSD and RW; ROI, right superior medial frontal gyrus.

#### 3.2.4 Robustness analyses with alternative network construction methods

To assess the stability of our graph-theoretical findings, we performed two additional sets of analyses while keeping all preprocessing and statistical procedures identical: (1) using an extended sparsity range of 0.05–0.25 (step = 0.01) on binary networks, and (2) using weighted networks across the original sparsity range (0.10–0.22). For weighted networks, we retained the Fisher-z transformed correlation coefficients as edge weights without binarization, and computed global and nodal metrics using the same AUC approach. The results of these robustness checks are summarized in Table 5.

**Table 5:** Comparison of results across different network construction methods.

Metric	Original binary (Sparsity 0.10–0.22)	Extended binary (Sparsity 0.05–0.25)	Weighted (Sparsity 0.10–0.22)
Global metrics			
Clustering coefficient	*t* = 2.089, *p* = 0.044*	*t* = 1.573, *p* = 0.124	*t* = 2.391, *p* = 0.023*
Nodal metrics			
Degree centrality (R SFGmed)	*t* = 3.745, *p* = 0.0006*	*t* = 3.748, *p* = 0.0006^†^	*t* = 4.247, *p* = 0.00015*
Nodal efficiency (L FG)	*t* = −3.865, *p* = 0.0004*	*t* = −3.525, *p* = 0.0012^‡^	n.s.
Nodal efficiency (R SFGmed)	n.s.	n.s.	*t* = 4.036, *p* = 0.00028*

*FDR correction was applied separately for each metric family within each network construction method, controlling the false discovery rate at *q* < 0.05.

Significant after FDR correction (*p* < FDR-adjusted threshold).

^†^In the extended binary network, the *p*-value for R SFGmed degree centrality (0.0006) was the smallest among all 90 nodes, though it did not survive FDR correction (FDR threshold = 0.0005).

^‡^In the extended binary network, the *p*-value for L FG nodal efficiency (0.0012) was the smallest among all 90 nodes, though it did not survive FDR correction (FDR threshold = 0.0005).

For global metrics, the increased clustering coefficient under TSD remained significant after FDR correction in the weighted network analysis (*p* = 0.023) and showed a non-significant trend in the extended sparsity range (*p* = 0.124). The direction of change was consistent across all methods, supporting a tendency toward enhanced local segregation after TSD.

For nodal metrics, the increased degree centrality in the right superior medial frontal gyrus (SFGmed. *r*) was highly robust, surviving FDR correction in both the original binary (*p* = 0.0006) and weighted (*p* = 0.00015) analyses. In the extended binary network, although the *p*-value (0.0006) did not meet the FDR-corrected threshold (0.0005), it was the smallest *p*-value among all 90 nodes, confirming that this region remains the most prominent focus of topological alteration.

The decreased nodal efficiency in the left fusiform gyrus (FG. L) was significant after FDR correction in the original binary analysis (*p* = 0.0004) and showed a strong trend in the extended binary analysis (*p* = 0.0012), where it was the smallest *p*-value among all nodes. In the weighted network, this effect was not significant.

Notably, nodal efficiency in the right SFGmed, which was not significant in binary networks, emerged as significantly increased after FDR correction in the weighted analysis (*p* = 0.00028).

### 3.3 Correlations of age and neuropsychological changes with baseline network attributes and their changes after TSD

#### 3.3.1 Correlations between changes in neuropsychological test performance and network attributes (baseline and change scores)

Exploratory correlation analyses revealed several associations with notable effect sizes. For instance, the changes in NCT-A performance correlated positively with both the changes in hierarchy (*r* = 0.336, *p* = 0.045) and the hierarchy value measured in the TSD condition (*r* = 0.330, *p* = 0.05). Similarly, the changes in NCT-A performance was correlated with the changes in local efficiency (*r* = 0.409, *p* = 0.013) and with local efficiency in the TSD condition (*r* = 0.401, *p* = 0.015). The changes in DST performance was correlated with the changes in lambda (*r* = 0.339, *p* = 0.043). It was also correlated with several baseline (RW condition) network metrics: global network efficiency (*r* = 0.34, *p* = 0.043), lambda (*r* = −0.351, *p* = 0.036), and average path length (*r* = −0.332, *p* = 0.048). The changes in LTT performance was correlated with baseline global network efficiency (*r* = −0.333, *p* = 0.047) and with lambda in the TSD condition (*r* = 0.337, *p* = 0.044). Especially, the TSD clustering coefficient was positively correlated with the changed NCT-A/SDT performance (*r* = 0.341, *p* = 0.042; *r* = 0.411, *p* = 0.013), as clustering coefficient enhanced after TSD.

#### 3.3.2 Correlations between age and baseline network attributes or their changes after TSD

Significant correlations were observed between participant age and brain network attributes (Table 6). Specifically, in the RW condition, older age was associated with lower global network efficiency (*r* = −0.352, *p* = 0.035), higher normalized path length (*r* = 0.350, *p* = 0.036), and longer average path length (*r* = 0.390, *p* = 0.019). Regarding the changes induced by TSD (TSD value – RW value), older age was correlated with a greater increase in global network efficiency (*r* = 0.382, *p* = 0.021), a greater decrease in lambda (*r* = −0.389, *p* = 0.019), and a greater decrease in average path length (*r* = −0.400, *p* = 0.016).

**Table 6:** Spearman correlations between age or neuropsychological changes and network attributes.

Global network attributes	Age, changes of Neuropsychological test	Effect size (*r* [95% CI])	*p* value
Hierarchy (TSD)	NCT-A	0.330 [−0.01, 0.60]	0.05
Hierarchy (TSD-RW)	NCT-A	0.336 [0.01, 0.60]	0.045
Global Network Efficiency (RW)	Age	−0.352 [−0.62, −0.02]	0.035
	DST	0.340 [0.01, 0.60]	0.043
	LTT	−0.333 [−0.61, 0.00]	0.047
Global Network Efficiency (TSD-RW)	Age	0.382 [0.06, 0.63]	0.021
Local Network Efficiency (TSD)	NCT-A	0.401 [0.08, 0.65]	0.015
Local Network Efficiency (TSD-RW)	NCT-A	0.409 [0.09, 0.66]	0.013
Clustering Coefficient (TSD)	NCT-A	0.341 [0.01, 0.61]	0.042
Clustering Coefficient (TSD)	SDT	0.411 [0.09, 0.66]	0.013
Lambda (TSD)	LTT	0.337 [0.01, 0.60]	0.044
Lambda (RW)	Age	0.350 [0.02, 0.61]	0.036
	DST	−0.351 [−0.62, −0.02]	0.036
Lambda (TSD-RW)	Age	−0.389 [−0.65, −0.07]	0.019
	DST	0.339 [0.01, 0.60]	0.043
Average Path Length (RW)	Age	0.390 [0.07, 0.64]	0.019
	DST	−0.332 [−0.61, 0.00]	0.048
Average Path Length (TSD-RW)	Age	−0.400 [−0.66, −0.08]	0.016

Correlation analysis was assessed by Spearman correlation. NCT-A, Number Connection Test-A; DST, Digit Symbol Test; LTT, Line Tracing Test; SDT, Serial Dotting Test.

#### 3.3.3 Validation of brain–behavior associations using linear regression

To further validate the robustness of the brain–behavior correlations reported in Section 3.1, we performed linear regression analyses controlling for age and sex. The results are summarized in Table 7.

**Table 7:** Linear regression analyses validating key brain–behavior associations.

Dependent variable	Predictors	B	Std. Error	Beta	*t*	*p*-value	95% CI for B		Tolerance	VIF
							Lower	Upper		
ΔSDT	(Constant)	988.877	423.156		2.337	0.026	124.845	1852.909		
	aCp	1834.629	744.891	0.385	2.382	0.023	143.125	1834.629	0.892	1.121
	Age	−10.215	8.234	−0.192	−1.24	0.224	−27.013	6.583	0.892	1.121
	Sex	2.456	15.678	0.024	0.157	0.876	−29.478	34.39	0.892	1.121
ΔNCT-A	(Constant)	2.614	1.234		2.118	0.042	0.097	5.131		
	aCp	2.715	0.954	0.445	2.804	0.009	0.772	4.658	0.892	1.121
	Age	−0.042	0.035	−0.183	−1.2	0.239	−0.113	0.029	0.892	1.121
	Sex	0.127	0.167	0.116	0.76	0.453	−0.213	0.467	0.892	1.121
ΔLp	(Constant)	0.059	0.023		2.516	0.017	0.011	0.106		
	Age	−0.002	0.001	−0.45	−2.539	0.016	−0.003	0	0.8	1.25
	Sex	−0.004	0.006	−0.114	−0.643	0.525	−0.016	0.008	0.8	1.25

All models are linear regressions with *N* = 36. ΔSDT, change in Serial Dotting Test score; ΔNCT-A, change in Number Connection Test-A score; ΔLp, change in average path length (TSD minus RW); aCp, clustering coefficient under TSD condition. Covariates: Age (in years) and Sex (male = 1, female = 0). Unstandardized coefficients (B) and their 95% confidence intervals, standardized coefficients (β), *t*-values, and *p*-values are reported. Collinearity statistics (Tolerance and VIF) indicate no multicollinearity concerns (all VIF < 2).

The clustering coefficient under TSD (aCp) remained a significant predictor of the change in Serial Dotting Test (ΔSDT) and the change in Number Connection Test-A (ΔNCT-A) after adjusting for covariates (ΔSDT: *β* = 0.385, *p* = 0.023; ΔNCT-A: *β* = 0.445, *p* = 0.009). In addition, the change in average path length (ΔLp) was significantly predicted by age (*β* = −0.450, *p* = 0.016), consistent with the correlation analysis. All VIF values were below 2, indicating no multicollinearity concerns. These results confirm that the observed associations are not confounded by age or sex and support the reliability of our findings.

## 4 Discussion

Previous studies indicate that following a single night of recovery sleep after TSD, most neurobehavioral measures returned to pre-deprivation levels, with the exception of self-reported vigor (Yamazaki et al., 2021). Therefore, we scheduled the MRI and neuropsychological testing sessions for the TSD and RW phases to occur during two separate night shifts, the RW and TSD assessments were scheduled at the beginning and end of two separate night-shift cycles, respectively, ensuring they were a minimum of 2 weeks apart. It was worth noting that this study was significantly different from previous ones in that the subjects faced a huge workload and psychological pressure during TSD. Previous research has indicated that SD exerts its most noticeable effects on cognitive performance during tasks demanding sustained and high levels of attentional resources (Casagrande et al., 1997). Therefore, it was extremely necessary to explore the brain network and cognitive changes after TSD of medical staff.

In this study, we found the differences in the topology of the whole-brain functional network of medical staff after TSD using graph theory analysis. Correspondingly, the neuropsychological assessments revealed a deterioration in several cognitive domains following TSD. Moreover, significant correlations were observed between changes in network topology metrics and performance on neuropsychological tests. These associations may provide a mechanistic explanation for the diverse cognitive deficits observed following SD. Our findings align with the emerging paradigm of using neuroimaging biomarkers to capture individual vulnerability, as evidenced by large-cohort studies linking structural brain metrics to dimensional psychopathology (Kuang et al., 2023), underscoring the value of network topology as a functional counterpart to such structural signatures. Notably, recent large-scale investigations of environmental influences on brain development, such as those examining peer environments in adolescents, have similarly identified distinct patterns of FC that mediate behavioral outcomes (Liu et al., 2025), reinforcing the notion that network-level reorganization represents a common neural pathway through which environmental demands shape cognition and behavior.

As a high-frequency night shift condition, the significant impairment of executive function of medical staff under TSD has special warning significance, which may lead to delayed response or decision-making errors in clinical operations (Sugden et al., 2012).

As for brain network changes, in terms of global network indicators, both the TSD and RW conditions exhibited small-world network characteristics. But the higher clustering coefficient was found in the TSD condition, reflecting greater local interconnectivity and fault tolerance, possibly as a compensatory mechanism after TSD (Tian et al., 2024; Qi et al., 2021). This was different from previous laboratory experiment (Ning et al., 2022). And this difference might reflect the fact that brain networks could respond to the stress of TSD by enhancing local information processing, and this compensatory mechanism may be related to the neuroadaptive formation of long-term night shift training of medical staff. In terms of local network indicators, the degree centrality of the right superior medial frontal gyrus increased in the TSD condition, while the nodal efficiency of the left fusiform gyrus decreased. These regional changes align with established sleep deprivation literature, wherein the medial prefrontal cortex (encompassing the superior medial frontal gyrus) is linked to cognitive adaptation, and the fusiform gyrus is associated with vulnerable visual processing networks (Adam et al., 2017). The increase in degree centrality in the right superior medial frontal gyrus indicates a central role for this region in adapting to sleep loss, consistent with its involvement in cognitive resilience and emotional regulation (Mashour et al., 2022). This enhanced connectivity may help preserve cognitive functions in challenging conditions. The decreased nodal efficiency in the left fusiform gyrus aligned with findings that sleep deprivation impacts visual processing and memory networks (Alfredo Spagna et al., 2021), likely by disrupting stable neural transmission, which might provide a neural explanation for the image misjudgment phenomenon related to postoperative fatigue in medical staff. Notably, increased degree centrality in the right superior medial frontal gyrus and reduced nodal efficiency in the left fusiform gyrus may reflect a resource reallocation strategy: enhancing top-down attentional control while sacrificing perceptual processing (Stenson et al., 2022). This frontal-up, posterior-down pattern is consistent with broader models of sleep-deprived brain reorganization that highlight compensatory prefrontal recruitment alongside degradation of sensory processing efficiency (Adam et al., 2017). Meanwhile, FC is enhanced in multiple bilateral frontal and temporal regions using right superior medial frontal gyrus as ROI, which may reflect the brain’s realignment of neural activity patterns to maintain basic cognitive function during sleep deprivation (Sheth et al., 2021; Sullivan et al., 2018).

The robustness of our findings was further supported by complementary analyses using an extended sparsity range and weighted networks. The increased centrality of the right SFGmed was consistently observed across all analytical approaches, and it consistently yielded the smallest *p*-values among all nodes in both binary analyses, underscoring its role as a key hub in the brain’s response to TSD. Interestingly, weighted network analysis revealed an additional increase in nodal efficiency within the same region, suggesting that TSD not only enhances the region’s connectivity degree but also strengthens the efficiency of its existing connections. In contrast, the reduced efficiency of the left fusiform gyrus was significant in the original binary analysis and showed a strong trend in the extended range, but was not significant in weighted networks, indicating that this effect may be driven more by the loss of connections rather than a weakening of retained connections. These nuances highlight the complementary value of binary and weighted graph measures in characterizing TSD-induced brain reorganization.

Our fMRI study reveals whole-brain topological reorganization following TSD in medical staff. It is noteworthy that complementary modalities such as EEG and fNIRS also highlight prefrontal vulnerability to sleep loss. Verweij et al. (2014) demonstrated reduced local clustering and increased path length in prefrontal EEG networks after TSD, while Mukli et al. (2021) using fNIRS showed attenuated task-related connectivity changes in the frontal cortex. Our findings align with this evidence by identifying specific prefrontal and fusiform hubs altered under TSD, extending the understanding of sleep-deprived brain networks with anatomical specificity.

Furthermore, the brain network attributes of participants under baseline and TSD conditions, along with the alterations in these attributes before and after TSD, significantly impacted the variation in performance on neuropsychological tests around the TSD period, which suggested that there was a covariant relationship between the decrease in attention and the decrease in the efficiency of whole-brain information integration. It is noteworthy that the correlations with the largest effect sizes (e.g., |*r*| > 0.4) involved changes in local network efficiency and average path length, pointing to these specific topological properties as promising candidates for future investigation into individual cognitive vulnerability. These findings align with earlier research demonstrating that 24 h of TSD reduced the integration of brain network topology, leading to specific deficits in attentional function while sparing cognitive switching abilities (Pesoli et al., 2021). Importantly, the close coupling between network topology and cognitive performance observed here resonates with a growing body of large-scale research seeking to establish robust brain-behavior associations. For example, recent work in adolescents has shown that objectively measured behavioral states are linked to mental health outcomes through specific patterns of FC, reinforcing the notion that network metrics can capture individual differences in cognitive vulnerability (Yu et al., 2025). Our study extends this paradigm by applying it to the acute stressor of TSD in a high-risk occupational condition.

Particularly, the increase in the clustering coefficient was associated with the changes in performance on the NCT-A and the SDT after TSD. This enhancement in the clustering coefficient post-TSD suggests its role in modulating cognitive test performance. While the increase in the clustering coefficient might serve as a compensatory mechanism, it did not fully offset the deficits observed in NCT-A performance. Conversely, the compensation appears to be more effective in the SDT, potentially explaining the absence of a significant performance drop in this study.

This study showed that age was significantly correlated with baseline global network efficiency, lambda, and average path length. As age increasing, the global network efficiency of brain networks generally declined, while the average path length and lambda increased, which might reflect thinning of neural connections or reduced white matter integrity, and this deterioration was closely related to the deficits of cognitive functions (Bennett and Madden, 2014). However, we found that the correlations of age and the changes of global network efficiency, lambda, and average path length after TSD were opposite with baseline. This might reflect a reorganization of brain networks in response to cognitive impairment caused by TSD (Pan et al., 2023). Furthermore, some studies found that older people have lower FC between default mode network areas compared to younger people (Ferreira and Busatto, 2013; Dennis and Thompson, 2014). And another study indicated that increasing age was associated with decreasing segregation of functional brain systems (Chan et al., 2014). These conclusions demonstrated the significant effects of age on FC and brain network topology, especially in sleep-deprived state, and therefore it was necessary to implement differentiated scheduling strategies for them. The robustness of these brain–behavior associations was further confirmed by linear regression analyses controlling for age and sex (Table 7), which yielded results consistent with the correlation analyses and showed no multicollinearity issues.

The findings of this study point to several concrete directions for future research and potential clinical translation. First, the identified key network nodes, particularly the right superior medial frontal gyrus, represent candidate targets for neuromodulation interventions (e.g., transcranial magnetic or electrical stimulation) that could be tested for their efficacy in enhancing cognitive resilience during or after night shifts. Second, the correlation between baseline network properties and cognitive vulnerability suggests that resting-state fMRI or more accessible neurophysiological markers (e.g., EEG) could be explored for individualized risk assessment, helping to identify staff most susceptible to fatigue-related errors. Third, to move from association to mechanism, future longitudinal or interventional studies are needed to establish causality and to examine whether modulating these specific network properties can directly mitigate cognitive decline under TSD. Finally, these results underscore the necessity of developing evidence-based shift scheduling policies that account for the recovery time needed for functional brain networks, potentially advocating for longer intervals between consecutive night shifts or mandatory rest periods.

## 5 Limitation

First, this study was conducted in a real-world clinical setting, therefore the working intensity and environment of each medical staff are variable, which may affect the results. Second, participants’ activity states (awake or asleep) were based on self-report rather than objective verification. Third, although our sample included physicians, nurses, and technicians, the limited size of each professional subcondition precluded meaningful analysis of potential differential effects of TSD across roles. Future studies with larger, stratified samples are warranted to address this question. Fourth, this study only used various neuropsychological tests to assess the cognitive impairment of medical staff, and lacked more objective physiological parameters. Fifth, the present graph-theoretic analysis focused on binary networks within a conventional sparsity window, a methodological choice that aligns with numerous prior sleep-deprivation studies and facilitates direct comparison. Future investigations employing weighted network analyses could provide complementary insights into the strength, rather than merely the presence, of functional connections altered by TSD. Furthermore, the regions identified in our nodal analysis were derived from the same dataset. Their consistency with prior literature (Adam et al., 2017) supports their biological relevance, but future studies using independent cohorts are warranted. Finally, the brain-behavior correlation analyses were exploratory in nature. While we report effect sizes and confidence intervals to facilitate a more nuanced interpretation, these findings require validation in future confirmatory studies with pre-specified hypotheses and appropriate control for multiple comparisons. Consequently, while the study was adequately powered to detect the main within-subject effects of TSD on network topology, it may have been underpowered for these exploratory correlation analyses, particularly to detect smaller effect sizes. The use of Spearman correlation, rather than multivariable regression, was appropriate for this hypothesis-generating stage given our sample size; future studies with larger cohorts should employ more robust models to control for potential confounders.

## 6 Conclusion

In conclusion, TSD induced notable changes in both global and node-specific attributes of brain networks, suggesting adaptive mechanisms to preserve cognitive function. These findings highlight potential targets for interventions, such as transcranial magnetic stimulation or pharmacological approaches, to mitigate the adverse cognitive effects of TSD in high-demand environments.

## Data Availability

The raw data supporting the conclusions of this article will be made available by the authors, without undue reservation.

## Footnotes

1
https://www.nitrc.org/projects/gretna/
